# Hyperpolarization-activated cyclic nucleotide gated channels: a potential molecular link between epileptic seizures and Aβ generation in Alzheimer’s disease

**DOI:** 10.1186/1750-1326-7-50

**Published:** 2012-10-03

**Authors:** Yuhki Saito, Tsuyoshi Inoue, Gang Zhu, Naoki Kimura, Motohiro Okada, Masaki Nishimura, Nobuyuki Kimura, Shigeo Murayama, Sunao Kaneko, Ryuichi Shigemoto, Keiji Imoto, Toshiharu Suzuki

**Affiliations:** 1 Laboratory of Neuroscience, Graduate School of Pharmaceutical Sciences, Hokkaido University, Kita12-Nishi6, Kita-ku, Sapporo, 060-0812, Japan; 2 Laboratory of Neurobiophysics, Graduate School of Medicine, Dentistry and Pharmaceutical Sciences, Okayama University, Okayama, 700-8530, Japan; 3 Department of Psychiatry, The First Affiliated Hospital of China Medical University, Shenyang, 110001, China; 4 Division of Neuroscience, Graduate School of Medicine, Mie University, Tsu, 514-8507, Japan; 5 Molecular Neuroscience Research Center, Shiga University of Medical Science, Otsu, 520-2192, Japan; 6 Laboratory of Disease Control, Tsukuba Primate Research Center, National Institute of Biomedical Innovation, Tsukuba, 305-0843, Japan; 7 Department of Neuropathology, Tokyo Metropolitan Institute of Gerontology, Itabashi-ku, Tokyo, 173-0015, Japan; 8 Brain Bank for Aging Research, Tokyo Metropolitan Institute of Gerontology, Itabashi-ku, Tokyo, 173-0015, Japan; 9 Departments of Neuropsychiatry, Graduate School of Medicine, Hirosaki University, Hirosaki, 036-8562, Japan; 10 Division of Cerebral Structure, National Institute for Physiological Sciences, Okazaki, 444-8585, Japan; 11 Department of Information Physiology, National Institute for Physiological Sciences, Okazaki, 444-8787, Japan

## Abstract

**Background:**

One of the best-characterized causative factors of Alzheimer’s disease (AD) is the generation of amyloid-β peptide (Aβ). AD subjects are at high risk of epileptic seizures accompanied by aberrant neuronal excitability, which in itself enhances Aβ generation. However, the molecular linkage between epileptic seizures and Aβ generation in AD remains unclear.

**Results:**

X11 and X11-like (X11L) gene knockout mice suffered from epileptic seizures, along with a malfunction of hyperpolarization-activated cyclic nucleotide gated (HCN) channels. Genetic ablation of HCN1 in mice and HCN1 channel blockage in cultured Neuro2a (N2a) cells enhanced Aβ generation. Interestingly, HCN1 levels dramatically decreased in the temporal lobe of cynomolgus monkeys (*Macaca fascicularis*) during aging and were significantly diminished in the temporal lobe of sporadic AD patients.

**Conclusion:**

Because HCN1 associates with amyloid-β precursor protein (APP) and X11/X11L in the brain, genetic deficiency of X11/X11L may induce aberrant HCN1 distribution along with epilepsy. Moreover, the reduction in HCN1 levels in aged primates may contribute to augmented Aβ generation. Taken together, HCN1 is proposed to play an important role in the molecular linkage between epileptic seizures and Aβ generation, and in the aggravation of sporadic AD.

## Background

Alzheimer's disease (AD) is characterized by progressive memory impairment, which accompanies aging. Genetic and biochemical studies show that the production of amyloid-β peptide (Aβ) largely contributes to the etiology of AD [1]. Aβ is generated from amyloid-β precursor protein (APP) by β- and γ-cleavage of the latter.

The risk of seizure activity is particularly high in AD patients, with an 87-fold increase in subjects with early-onset dementia compared with an age-matched reference population [2-7]. Factors linking seizure activity to Aβ generation in AD patients remain unclear, although epilepsy is believed to result from abnormal regulation of neuronal excitability, which favors hypersynchrony. In addition, increased neuronal activity enhances Aβ production from APP [8-10].

Hyperpolarization-activated cyclic nucleotide gated HCN channels 1–4 (HCN1–4) conduct inward, depolarizing mixed Na^+^/K^+^ currents and thereby control resting membrane potential, dendritic integration, synaptic transmission, and rhythmic activity in cardiac pacemaker cells and spontaneous firing neurons [11]. Dysregulation of these channels and their hyperpolarization-activated (Ih) currents is strongly implicated in various experimental animal models of epilepsy, as well as in human epilepsy patients [12]. Furthermore, HCN2 co-assembles with the X11-like (X11L) protein [13], which is a metabolic regulator of APP processing [14].

X11 proteins (X11s) comprise a family of three evolutionarily conserved molecules (X11/X11α/Mint1, X11L/X11β/Mint2, and X11L2/X11γ/Mint3). These proteins bind to the cytoplasmic region of APP in cultured cells and suppress its metabolism [15,16]. Moreover, the metabolism of overexpressed human APP (hAPP) is suppressed in X11 and X11L transgenic mice, along with the generation of Aβ [17-19]. On the other hand, mutant mice lacking X11L (X11^+/+^/X11L^-/-^ mice) or both X11 and X11L (X11^-/-^/X11L^-/-^ mice) facilitate amyloidogenic metabolism of endogenous murine APP and exogenous hAPP, including Aβ generation [20-22]. Therefore, inactivation of X11/X11L clearly increases the production of Aβ, potentially contributing to the pathology of AD.

Here, we report that i) X11^-/-^/X11L^-/-^ mice suffer from spontaneous epileptic seizures along with malfunction of HCN channel activity; ii) HCN1 can form a complex with APP and X11 or X11L in the murine brain; iii) HCN1^-/-^ gene knockout mice show enhanced Aβ generation; iv) overexpression of HCN1 in Neuro2a (N2a) cells decreases Aβ generation, whereas blockage of HCN1 channel activity in N2a cells restores the level of Aβ production; v) the level of HCN1 diminishes significantly in the temporal cortex of cynomolgus monkeys (*Macaca fascicularis*) during aging; and vi) HCN1 levels are significantly reduced in the brains of sporadic AD patients compared with the brains of age-matched healthy subjects.

Given the previous reports and our current observations, we hypothesize that X11 and X11L play an important role in the modulation of HCN channel function, the dysregulation of which correlates with epilepsy. We further hypothesize that the impairment of HCN channels, and in particular HCN1, accompanies with the aberrant production of Aβ, which manifests as neurotoxicity. Thus, HCN1 together with X11 and X11L may provide a molecular link between seizure activity and Aβ generation in AD patients.

## Results

### Spontaneous epileptic seizures caused by X11 and X11L gene deficiency

**Figure 1:**
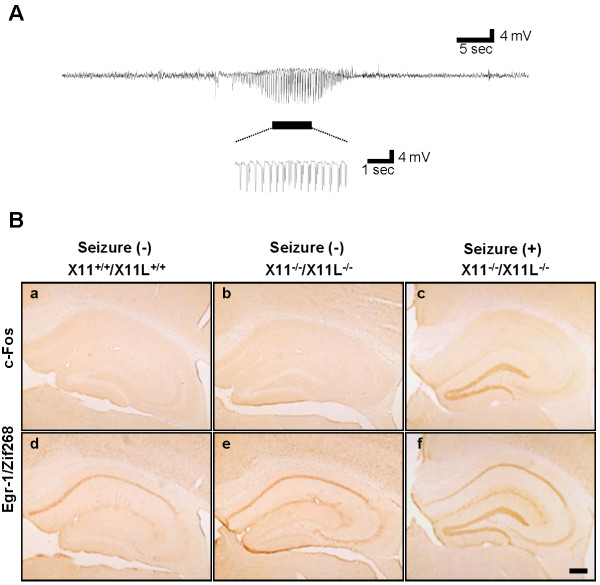
**Electrocorticogram recording and histological changes associated with epileptic seizures in X11**^**-/-**^**/X11L**^**-/- **^**mice.** (***A***) An electrocorticogram recorded in 13-week-old X11^-/-^/X11L^-/-^ mice (n = 4). Epileptic discharges are underlined. (***B***) c-Fos (*a, b, c*) and Egr-1/zif268 (*d, e, f*) immunoreactivity in the hippocampus of X11^+/+^/X11L^+/+^ (*a, d*) and X11^-/-^/X11L^-/-^ (*b, c, e, f*) mice under steady-state conditions (*a, b, d, e*) and at 90 min after an epileptic episode (*c, f*). Scale bar, 200 μm.

Electrocorticograms were recorded in X11^+/+^/X11L^+/+^ (wild type), X11^+/+^/X11L^-/-^, X11^-/-^/X11L^+/+^, and X11^-/-^/X11L^-/-^ mice. We found that X11^-/-^/X11L^-/-^ mice suffered from spontaneous epileptic seizures at the age of 13 weeks and over (detailed results are provided in Additional file 1: Figure S1 and Additional file 2: Movies S1, Additional file 3: Movies S2 and Additional file 4: Movies S3). Three out of four X11^-/-^/X11L^-/-^ mice showed an abnormal electrocorticogram recording within 48 h, namely, the presence of epileptic discharge, which were never observed in X11^+/+^/X11L^+/+^, X11^+/+^/X11L^-/-^, or X11^-/-^/X11L^+/+^ mice (Figure 1, Additional file 1: Figure S1 and Additional file 4: Movie S3). Subsets of X11^-/-^/X11L^-/-^ mice went into status epilepticus and died.

Seizures are often associated with the augmented expression of immediate-early genes in neurons [23]. We first asked whether such gene activation was observed in X11^-/-^/X11L^-/-^ mice following epileptic seizures and investigated the involvement of specific brain regions in seizure activity. Brain tissue sections from X11^-/-^/X11L^-/-^ mice were immunostained for c-Fos, a calcium-dependent immediate-early gene product, and Egr-1/Zif268, an early growth response transcription factor, within 90 min of a seizure event. The brains of these mice showed enhanced expression of both c-Fos and Egr-1/Zif268 in the dentate gyrus (DG) granule cells compared with the brains of X11^+/+^/X11L^+/+^ and X11^-/-^/X11L^-/-^ mice (Figure 1B). However, we cannot rule out a possibility that subclinical discharges without aberrant behavior may cause the enhanced expression of c-Fos and Egr-1/Zif268. Thus, a deficiency in both X11 and X11L may cause abnormal, seizure-associated neuronal activity and subsequent alterations in protein expression in the hippocampal formation.

### Reduction of Ih currents in entorhinal cortex (EC) layer II of X11^-/-^/X11L^-/-^ mice

**Figure 2:**
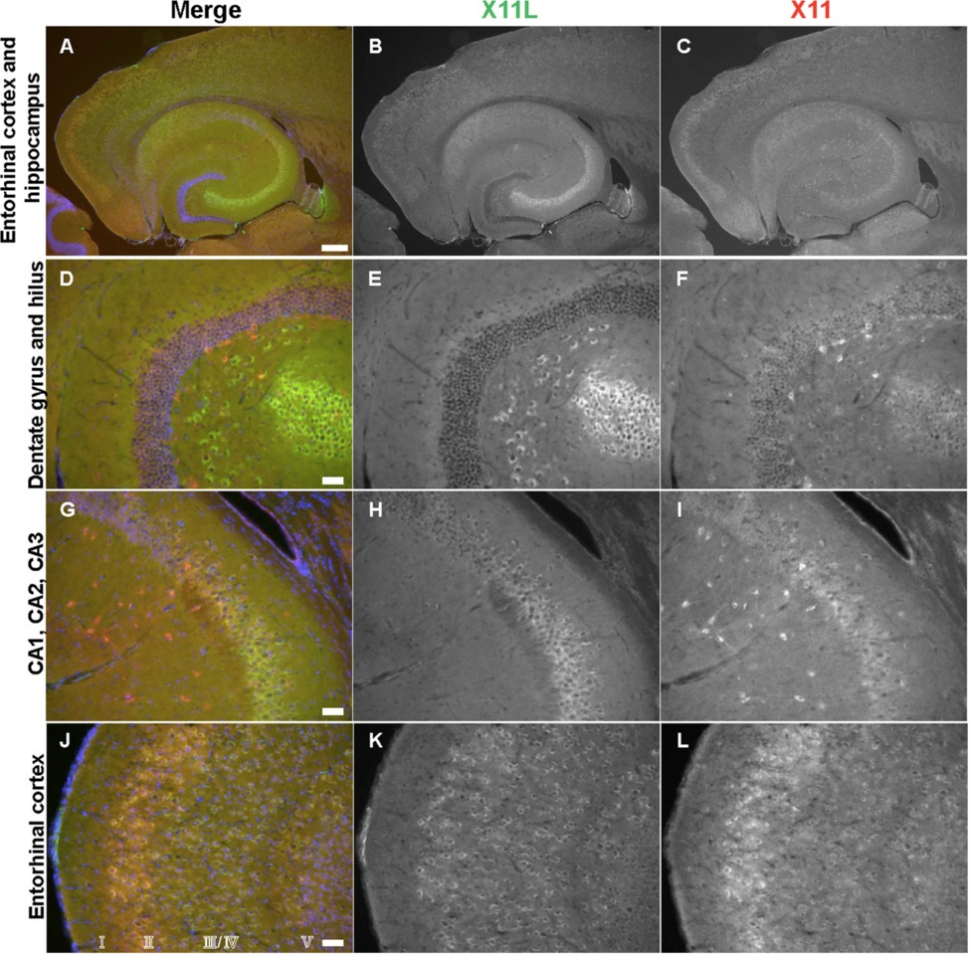
**Expression of X11 and X11L in the 13-week-old mouse brain.** Representative images of horizontal brain sections from 13-week-old X11^+/+^/X11L^+/+^ mice were stained for X11 (***C, F, I, L***) and X11L (***B, E, H, K***). Immunohistochemistry was performed with anti-X11 polyclonal rabbit IgG followed by donkey anti-mouse IgG conjugated to Alexa Fluor 488, and anti-X11L monoclonal mouse IgG followed by donkey anti-rabbit IgG conjugated to Cy3. Merged images are shown in the *left panels* (***A, D, G, J***). Blue signals indicate nuclei counter-stained with DAPI. Scale bars, 300 μm (***A–C***), 50 μm (***D–L***).

Spontaneous epileptic seizures were observed in mice when both X11 and X11L genes were deficient (Figure 1A, Additional file 1: Figure S1 and additional movies). Because the DG granule cells of X11^-/-^/X11L^-/-^ mice showed augmented expression of c-Fos and Egr-1/Zif268 following seizure activity, we next performed a detailed examination of the expression of both proteins in hippocampal neurons in 13-week-old murine brains (Figure 2).

Distinct expression patterns of X11 and X11L were observed in the hippocampus of wild type mice. X11L was expressed mainly in the pyramidal neurons of the CA1–3 region (Figure 2B, E, H), whereas X11 was expressed in other types of interneurons (Figure 2C, F, I). These observations coincide with our previous report of X11s expression in aged wild type mice [21]. Unlike c-Fos and Egr-1/Zif268 in the double mutant mouse, X11 and X11L were not expressed in DG granule cells (Figure 2D–F). However, both X11 and X11L were strongly co-expressed in EC layer II (Figure 2J–L), which projects axons primarily to the granule cells of the DG [24]. Furthermore, both HCN1 and HCN2 are expressed in EC layer II [25]. Given that HCN1^-/-^ mice show enhanced seizure susceptibility and that HCN2^-/-^ mice suffer from absence seizures [26,27], we next focused our investigations on the alteration of Ih currents associated with HCN channels in EC layer II in X11^-/-^/X11L^-/-^ mice.

**Figure 3:**
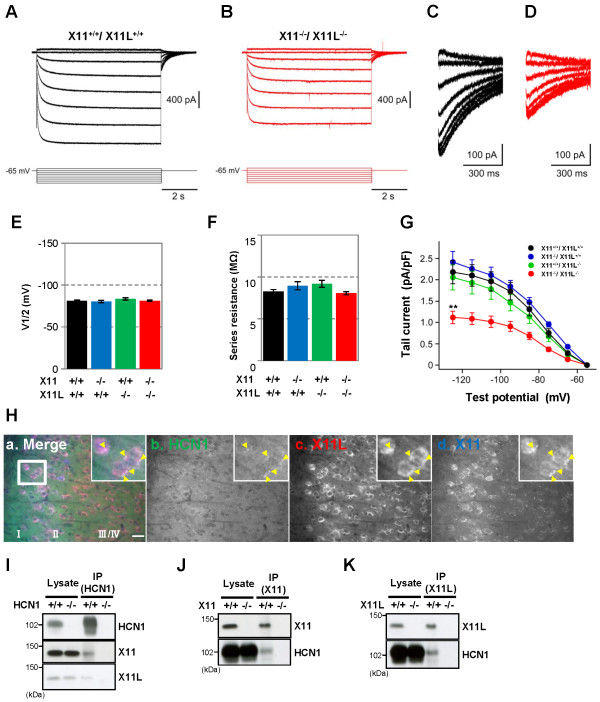
**Reduction of Ih currents in entorhinal cortex layer II neurons of X11**^**-/-**^**/X11L**^**-/- **^**mice. (*A, B*)** Representative electrophysiological recordings of Ih currents from EC layer II neurons in X11^+/+^/X11L^**+/+**^**(*A*)** and X11^-/-^/X11L^-/-^**(*B*)** mice. (***C, D***) Tail currents of X11^+/+^/X11L^+/+^ (***C***) and X11^-/-^/X11L^-/-^ (***D***) mice. (***E***) Quantitative data of the voltage, whereby the current is half-activated (V1/2). Statistical analysis was performed using one-way analysis of variance followed by Tukey’s multiple comparison test (mean ± SEM (mV); five slices from two X11^+/+^/X11L^+/+^ mice, n = 10; four slices from two X11^-/-^/X11L^+/+^, X11^+/+^/X11L^-/-^, and X11^-/-^/X11L^-/-^ mice, n = 9). (***F***) Quantitative data of series resistance. Statistical analysis was performed as described (mean ± SEM (MΩ); five slices from two X11^+/+^/X11L^+/+^ mice, n = 10; four slices from two X11^-/-^/X11L^+/+^, X11^+/+^/X11L^-/-^, and X11^-/-^/X11L^-/-^ mice, n = 9). (***G***) Summary of the current density of the tail currents. Statistical analysis was performed as described (mean ± SEM (pA/pF); five slices from two X11^+/+^/X11L^+/+^ mice, n = 10; four slices from two X11^-/-^/X11L^+/+^, X11^+/+^/X11L^-/-^, and X11^-/-^/X11L^-/-^ mice, n = 9; **p < 0.01). (***H***) Representative images of the HCN1-X11-X11L complex in EC layer II neurons of 13-week-old X11^+/+^/X11L^+/+^ mice (a, merge; b HCN1 (green); c, X11L (red); d, X11(blue)). Scale bar, 20 μm. (***I−K***) Co-immunoprecipitation of HCN1-X11s complexes from the 13-week-old X11^+/+^/X11L^+/+^ murine cortex. HCN1^-/-^ (***I***), X11^-/-^ (***J***), or X11L^-/-^ (***K***) mice were used as controls. Brain lysates were immunoprecipitated with anti-HCN1 (***I***), anti-X11 (***J***), and anti-X11L/Mint2 (***K***) antibodies. Immunocomplexes were detected by immunoblotting.

Horizontal brain slices that included the EC and the hippocampus were prepared from 12–14-week-old X11^+/+^/X11L^+/+^, X11^+/+^/X11L^-/-^, X11^-/-^/X11L^+/+^, and X11^-/-^/X11L^-/-^ mice. EC layer II neurons were then subjected to electrophysiological analysis, and Ih currents from HCN channels were recorded (Figure 3 and Additional file 1: Figure S2). The mice used in the electrophysiological study were seizure-naïve, at least without over behavioral manifestations, and showed comparable levels of HCN1 channels in the EC (Additional file 1: Figure S3). Similar to a previous report [28], hyperpolarizing voltage steps activated a large Ih current in EC layer II cells of X11^+/+^/X11L^+/+^ mice (Figure 3A and C). By contrast, the Ih current was dramatically reduced in X11^-/-^/X11L^-/-^ mice relative to that in X11^+/+^/X11L^+/+^ mice (Figure 3B and D); however, no significant alterations were observed for the V1/2 (mean ± SEM, X11^+/+^/X11L^+/+^: −81.1±1.1 mV; X11^-/-^/X11L^+/+^: −80.3±1.3 mV; X11^+/+^/X11L^-/-^: −83.5±1.4 mV; X11^-/-^/X11L^-/-^: −81.3±0.9 mV) (Figure 3E) or the series resistance (mean ± SEM, X11^+/+^/X11L^+/+^: 8.3±0.2 MΩ; X11^-/-^/X11L^+/+^: 8.9±0.5 MΩ; X11^+/+^/X11L^-/-^: 9.2±0.4 MΩ; X11^-/-^/X11L^-/-^: 8.0±0.2 MΩ) (Figure 3F). Quantitative analysis (Figure 3G) revealed that the density of the Ih current was also significantly reduced in X11^-/-^/X11L^-/-^ mice (1.12±0.15 pA/pF, n = 9; *p* < 0.01) compared with that in X11^+/+^/X11L^+/+^ mice (2.18±0.27 pA/pF, n = 10), but did not change significantly in X11^-/-^/X11L^+/+^ mice (2.41±0.25 pA/pF, n = 9, *p* > 0.05) or in X11^+/+^/X11L^-/-^ mice (2.05±0.29 pA/pF, n = 9, *p* > 0.05). Thus, genetic ablation of X11 and X11L together had a profound impact on the Ih current in the EC layer II of the double knockout mice. These results correlate with the observation that X11^-/-^/X11L^-/-^ mice, but not X11^+/+^/X11L^+/+^, X11^+/+^/X11L^-/-^, or X11^-/-^/X11L^+/+^ mice, are susceptible to spontaneous epileptic seizures.

In EC layer II, the dominant HCN subtype is HCN1 [28]. We found that HCN1, X11, and X11L were colocalized in EC layer II neurons (Figure 3H) and apparently formed a complex in the brain (Figure 3I–K). The colocalization of these molecules was observed in a region surrounding the neuronal nucleus (Figure 3H), consistent with the location of the Golgi apparatus. Because X11 and X11L are largely localized in the Golgi apparatus and function in the trafficking of membrane proteins [29,30], the deletion of X11 and X11L may disturb intracellular localization of HCN channels (Additional file 1: Figure S4). While the localization of the channel likely affects its function, we cannot rule out the possibility that X11 and X11L directly regulate HCN1 function as well.

### Enhanced Aβ generation according to HCN1 dysfunction

**Figure 4:**
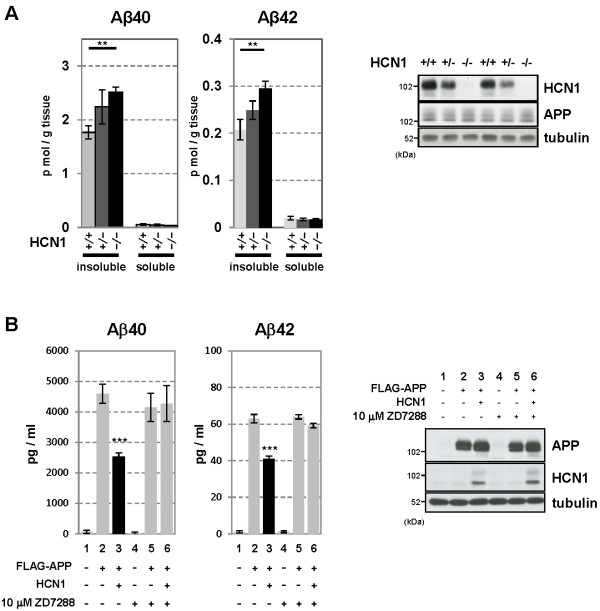
**Functional deficits in the HCN1 channel facilitate Aβ generation.** (***A***) Quantification of endogenous murine Aβ40 and Aβ42 in the cortex of 4-month-old HCN1^+/+^, HCN1^+/-^, and HCN1^-/-^ mice by using the sELISA system (left panels). Statistical analysis was performed using the two-tailed Mann–Whitney *U*-test (average ± SEM, n = 5, **p < 0.01). (***B***) Quantification of Aβ40 and Aβ42 secreted into the culture medium by N2a cells by using the sELISA system. FLAG-APP or FLAG-APP and HCN1 were transiently overexpressed in the cells with (+) or without (−) 10 μM ZD7288 (left panels). Statistical analysis was performed using one-way analysis of variance followed by Tukey’s multiple comparison test (average ± SEM, n = 4, ***p < 0.001). Expression levels of APP and HCN1 was confirmed by immunoblotting (right panels in ***A ***and ***B***).

The EC is one of the most vulnerable brain regions in AD [31], and it is well-known that synaptic activity such as that mediated by HCN channels can regulate Aβ generation [8-10]. Therefore, we examined whether HCN channel impairment involved in the aberrant production of Aβ. We first quantified the levels of endogenous Aβ40 and Aβ42 in HCN1^-/-^ mouse brains. Aβ40 and Aβ42 were both significantly increased in the cortex of HCN1^-/-^ mice compared with HCN1^+/+^ mice (average ± SEM, Aβ40: n = 5, *p* = 0.0037; Aβ42: n = 5, *p* = 0.0055) (Figure 4A). The magnitude of the increase in Aβ40 and Aβ42 was inversely proportional to the level of HCN1 gene expression (Figure 4A, left panel), while APP protein levels were comparable in HCN1^+/+^, HCN1^+/-^, and HCN1^-/-^ mice (Figure 4A, right panel).

To confirm whether the increase in Aβ generation in HCN1^-/-^ mice depends on the decrease in HCN channel activity, we used N2a cells that transiently overexpressed FLAG-APP and HCN1 (Figure 4B). Overexpression of HCN1 significantly reduced the generation of Aβ40 and Aβ42 (compare column 2 with column 3 for each Aβ peptide). The Aβ levels were restored by adding ZD7288, a selective inhibitor of HCN channel activity (compare lanes 3 through 6). Although we have not confirmed the blockage of channel activity electrophysiologically, ZD7288 had no effect on Aβ levels in cells without HCN1 expression (column 5), and APP expression was not affected by either the presence of HCN1 or by the administration of ZD7288 (Figure 4B, right panel). Furthermore, administration of ZD7288 did not influence the interaction of HCN1 with APP (Additional file 1: Figure S5). These results suggest that the suppression of Aβ generation in HCN1-overexpressing cells probably depends on channel activity (Figure 4B), in agreement with the *in vivo* observation that the brains of mice lacking the HCN1 gene and with impaired HCN channel activity (Figure 3B, D, and G) demonstrated increased Aβ generation (Figure 4A).

### Association of HCN1 with APP *in vivo* and *in vitro*

**Figure 5:**
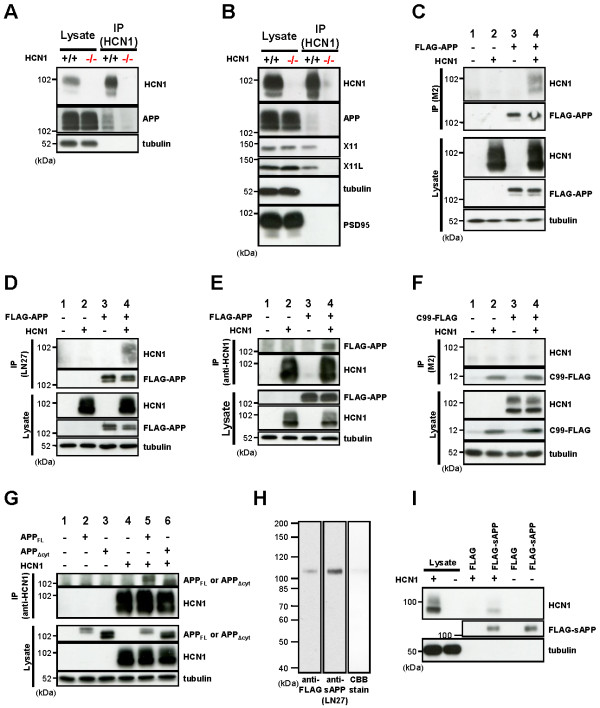
**APP and HCN1 form a molecular complex *in vivo *and *in vitro*.** (***A***) Co-immunoprecipitation of the HCN1-APP complex from the wild type and HCN1^-/-^murine cortex. Brain lysates were immunoprecipitated with an anti-HCN1 antibody. Immunocomplexes were detected by immunoblotting. (***B***) Co-immunoprecipitation of HCN1-APP, -X11 and -X11L complexes from the EC-rich cortex (see Additional file 1: Figure S2). Brain lysates were immunoprecipitated with an anti-HCN1 antibody. Immunocomplexes were detected by immunoblotting. (***C–G***) Co-immunoprecipitation of the HCN1-APP complex from N2a cells transiently overexpressing FLAG-APP and murine HCN1. (***C–E***), Analysis of FLAG-APP and murine HCN1, (***F***) C99-FLAG and murine HCN1, and (***G***) APP_FL_ or APP_Δcyt_ and murine HCN1 immunocomplexes. To standardize the amount of plasmid, empty vector (−) was added to yield 1.2 μg of plasmid in total. Cell lysates were immunoprecipitated with anti-FLAG M2 (***C*** and *F*), anti-APP extracellular domain (LN27) (*D*), or anti-HCN1 (***E*** and ***G***) antibodies. Immunocomplexes were detected by immunoblotting. (***H***) Affinity purification of FLAG-sAPP secreted into the culture medium by N2a cells expressing FLAG-APP. The purification was performed using anti-FLAG M2 affinity beads. FLAG-sAPP were specifically detected by M2 and anti-APP extracellular domain (LN27) antibodies, and no contaminating bands were identified by CBB-staining. (***I***) Pull-down of HCN1 with affinity purified FLAG-sAPP prepared in (H). Lysates from wild type cells (−) and cells that transiently expressed HCN1 (+) were incubated with M2 affinity beads coupled with FLAG-tag or FLAG-sAPP. The complexes resulting from the pull-down assay were subjected to immunoblot analysis with anti-HCN1 antibody.

Increased synaptic activity enhances Aβ generation [8-10], and modulation of Aβ generation is not limited to alterations in HCN1 channel activity. Indeed, APP metabolism is thought to be largely regulated by APP-binding partners [14]. Therefore, we next explored the hypothesis that HCN1 might be involved in regulating APP metabolism via a physical interaction between the channel and APP. In support of this hypothesis, an anti-HCN1 antibody co-immunoprecipitated APP together with HCN1 from a lysate of wild type murine cortex (Figure 5A). The interaction seemed to be specific in that APP was not recovered from the cortical lysate of HCN1^-/-^ mice. The association between APP and HCN1 was next confirmed in the EC. Using EC-rich brain samples isolated from HCN1^+/+^ and HCN1^-/-^ mice (Additional file 1: Figure S3A), a co-immunoprecipitation assay was performed with an anti-HCN1 antibody, and the immunoprecipitates were analyzed with the indicated antibodies (Figure 5B). Along with X11 and X11L, APP was co-immunoprecipitated with HCN1 in EC-rich brain samples of wild type mice (Figure 5B), suggesting that HCN1 can complex with APP and X11/X11L *in vivo*. These results are in agreement with the co-localization results of HCN1, X11, and X11L in the wild type cortex shown above (Figure 3H–K). Tubulin and PSD95 (postsynaptic density protein 95) were not detected in the immunocomplex, indicating the specific association of APP and X11/X11L with HCN1.

To show whether HCN1 directly binds to APP without mediation by X11/X11L, FLAG-APP and HCN1 were transiently expressed in N2a cells and a co-immunoprecipitation assay was performed. The anti-FLAG M2 antibody immunoprecipitated HCN1 along with FLAG-APP (Figure 5C). An anti-hAPP extracellular domain antibody (LN27) also recovered HCN1 (Figure 5D), and anti-HCN1 antibody recovered FLAG-APP (Figure 5E).

APP is a type I transmembrane protein composed of a large extracellular (luminal) domain of 596 amino acids and a small intracellular domain of 47 amino acids. On the other hand, HCN1 is six-transmembrane protein with short extracellular sequences between transmembrane regions one and two, three and four, and five and six; and long intracellular domains within the amino- and carboxyl-terminal regions of the protein. To determine the region of APP that binds to HCN1, we performed co-immunoprecipitation assays using APP deletion mutants (C99-FLAG, which largely lacks the extracellular domain of APP and in which FLAG is fused to the carboxyl terminal region of APP; and APP_Δcyt_, which lacks the 43- amino acid carboxyl terminal region of APP) (Figure 5F, G). The results of this assay indicated that HCN1 was not co-immunoprecipitated with C99-FLAG (Figure 5F), whereas APP_Δcyt_ was co-immunoprecipitated with HCN1 (Figure 5G).

Next, we performed an *in vitro* pull-down assay with FLAG-soluble APP (FLAG-sAPP, consisting of the extracellular domain of APP cleaved at the α- and/or β-cleavage sites). FLAG-sAPP was purified with affinity beads (anti-FLAG M2 affinity gel) from the culture medium of N2a cells expressing FLAG-APP (Figure 5H) and then incubated with lysates of N2a cells that expressed HCN1. HCN1 bound to FLAG-sAPP, but not to FLAG-tag alone (Figure 5I). Taken together, the results shown in Figure 5 indicate that HCN1 associates with APP through its extracellular (luminal) domain.

Hence, HCN1 apparently interacts with the extracellular domain of APP (Figure 5) and with both X11 and X11L in the cytoplasm (Figure 3H–K, Figure 5B). This suggests that the HCN1 channel might form a ternary complex with APP and either X11 or X11L to regulate Aβ generation. However, the detailed molecular regulation of complex formation remains to be determined.

### Age- and AD state-dependent HCN disruption in the temporal cortex (superior temporal gyrus) of cynomolgus monkeys and sporadic AD patients

**Figure 6:**
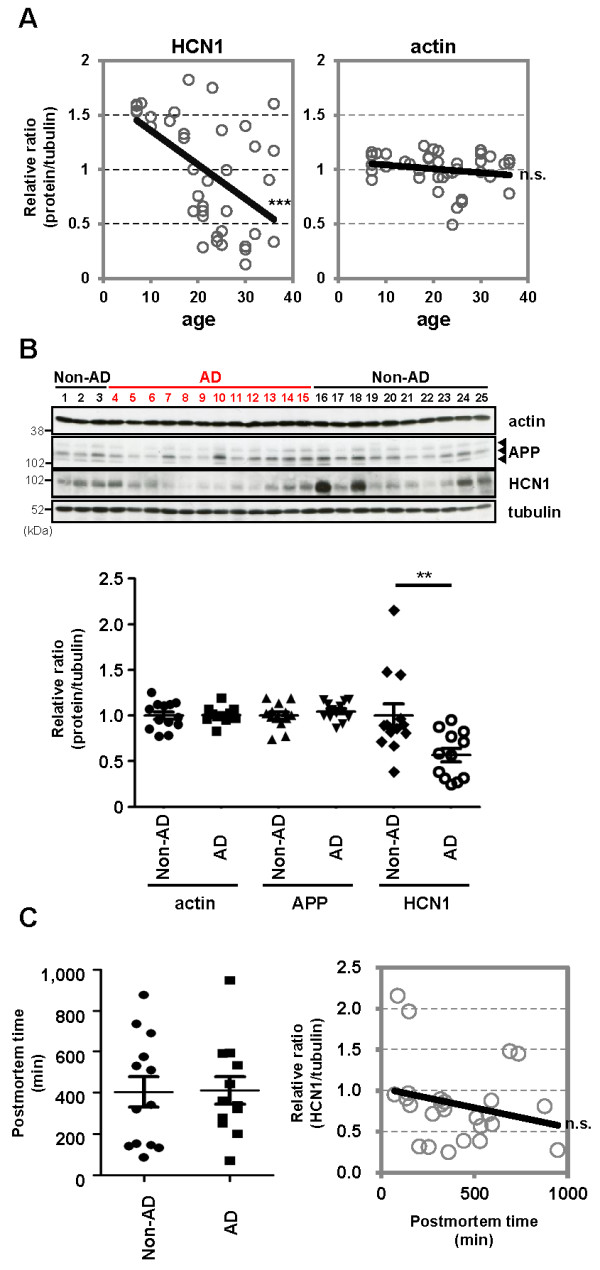
**HCN1 levels decrease in a age- and AD-dependent manner.** (***A***) Quantification of the amount of HCN1 and actin in the brain (superior temporal gyrus) of 4–37-year-old cynomolgus monkeys by immunoblotting. Statistical analysis was performed using the two-tailed Pearson's correlation coefficient (HCN1: n = 39, r = −0.5363, ***p < 0.001; actin: n = 39, r = −0.1981, p > 0.05). (***B***) Quantification of the amount of HCN1, APP, actin, and tubulin in age-matched AD and non-AD postmortem brains (superior temporal gyrus) by immunoblotting. Statistical analysis was performed using the two-tailed Mann–Whitney *U*-test (non-AD: n = 13, AD: n = 12, **p < 0.01). (***C***) Comparison of postmortem time between non-AD and AD samples. Statistical analysis was performed using the two-tailed Mann–Whitney *U*-test (non-AD: n = 13, AD: n = 12, p = 0.8066). The correlation between postmortem time and HCN1 level was analyzed. Statistical analysis was performed using the two-tailed Pearson's correlation coefficient (n = 39, r = −0.2416, p = 0.2446). Descriptions of all subjects are provided in Table 1.

Advanced age is the greatest risk factor for AD. To examine the relationship between aging and HCN1 levels, we quantified the amount of HCN1, Aβ, APP, and actin in freshly frozen brain tissues (superior temporal gyrus) from cynomolgus monkeys of various ages (Figure 6A and Additional file 1: Figure S6). Senile plaques and neurofibrillary tangles spontaneously appear in the brains of cynomolgus monkeys with advancing age [32,33], and the amino acid sequence of Aβ in cynomolgus monkeys is identical to that in humans [34]. Thus, we hypothesized that the cynomolgus monkey would be a useful animal model for the investigating the relationship between aging and AD pathology. Significant negative correlations were found between HCN1 levels and age (n = 39, r = −0.5363, *p* = 0.0004) (Figure 6A, left), between HCN1 and APP levels (n = 39, r = −0.3796, *p* = 0.0086) (Additional file 1: Figure S6B), between HCN1 and TBS-insoluble Aβ40 levels (n = 39, r = −0.2878, *p* = 0.0421) (Additional file 1: Figure S6C and D), and between HCN1 and Tris buffered saline (TBS)-insoluble Aβ42 levels (n = 39, r = −0.2913, *p* = 0.0401) (Additional file 1: Figure S6E and F). A significant positive correlation was found between age and APP level (n = 39, r = 0.8156, *p* < 0.0001) (Additional file 1: Figure S6A), and a significant weak-positive correlation was found between APP and TBS-insoluble Aβ42 levels (n = 39, r = 0.3714, *p* = 0.0236) (Additional file 1: Figure S6H). However, no correlation was found between age and actin level (n = 39, r = −0.1981, *p* = 0.2266) (Figure 6A, right) or between APP and TBS-insoluble Aβ40 levels (n = 39, r = 0.2993, *p* = 0.072) (Additional file 1: Figure S6G).

Finally, we examined the possibility of altered HCN1 levels in human AD brain specimens obtained at autopsy (Figure 6B and Table 1). The postmortem time was not significantly different for the AD and non-AD brain samples used in this study (non-AD: n = 13, AD: n = 12, *p* = 0.8066), and no significant decrease in HCN1 levels related to postmortem time was observed (n = 25, r = −0.2416, *p* = 0.2446) (Figure 6C and Table 1). Of great relevance to this study, the amount of HCN1 was significantly reduced in AD brains (superior temporal gyrus) compared with that in age-matched control brains (non-AD: n = 13, AD: n = 12, *p* = 0.0083), while the levels of APP and actin were not significantly altered (Figure 6B and Table 1). These results suggest that the reduction in HCN1 expression that occurs with age (Figure 6A) may be involved in the aggravation of the pathology of AD.

**Table 1:** **Summary of subject information presented in Figure**6**B and C**

**Sample no.**	**Subject**	**Age**	**Sex**	**Post mortem time (min)**	**Braak stage**	**Seizure**	**Relative ratio (protein/tubulin)**		
							**Actin**	**APP**	**HCN1**
1	Normal	84	F	877	1	-	0.929	1.192	0.809
2	Normal	88	F	144	1	-	0.856	1.046	0.859
3	Normal	70	M	340	0.5	-	0.955	1.189	0.859
4	AD	84	M	71	4	-	0.950	1.171	0.952
5	AD	70	M	276	4	-	0.829	0.986	0.716
6	AD	74	M	322	5	-	0.961	0.990	0.827
7	AD	75	M	594	6	-	1.069	1.176	0.589
8	AD	76	M	947	6	+	1.190	1.167	0.273
9	AD	79	F	203	5	-	1.019	0.913	0.317
10	AD	81	M	360	6	-	0.971	0.862	0.247
11	AD	81	F	442	5	-	0.971	0.862	0.385
12	AD	82	F	254	6	-	0.988	1.029	0.313
13	AD	82	F	533	5	-	0.988	1.001	0.566
14	AD	82	F	338	4	-	1.028	1.063	0.770
15	AD	84	M	590	5	-	1.071	1.128	0.876
16	Normal	78	F	87	2	-	1.108	1.138	2.153
17	Normal	81	M	134	1	-	1.122	0.971	0.902
18	Normal	82	F	148	1	-	1.178	1.109	1.963
19	Normal	82	F	155	2	-	1.141	1.026	0.821
20	Normal	80	M	317	2	-	1.254	1.037	0.893
21	Normal	80	M	510	2	-	1.071	0.778	0.669
22	Normal	82	M	530	1	-	0.979	0.916	0.384
23	Normal	78	M	575	1	-	0.905	1.007	0.713
24	Normal	78	M	690	1	-	0.784	0.986	1.479
25	Normal	82	M	736	1	-	0.774	0.743	1.448

Postmortem brain samples were obtained from the Brain Bank for Aging Research, Tokyo Metropolitan Institute of Gerontology. Experimental procedures were approved by the appropriate ethical boards at each institute. Autopsies were performed with written informed consent from the patients or their relatives. The clinical diagnosis of AD was based on two major criteria: the Diagnostic and Statistical Manual of Mental Disorders: 4th Edition (DSM-IV) and the National Institute of Neurological and Communicational Disorders and Stroke-Alzheimer's Disease and Related Disorders Association (NINCDS-ADRDA). The neuropathological diagnosis of AD was made using Consortium to Establish a Registry for Alzheimer’s Disease criteria (average age at death, 79.2 ± 4.4 years). Control brains were obtained from age-matched individuals with no history of neurological or psychiatric illness (average age at death, 80.4 ± 4.2 years). Subjects, age, gender, postmortem time, Braak stage, seizure history, and relative protein/tubulin ratios are indicated.

## Discussion

X11 and X11L are well-characterized neural adaptor proteins that regulate the trafficking and metabolism of APP [14]. Many reports indicate that X11s bind to APP and suppress Aβ generation *in vitro* and *in vivo*[14-22]. Furthermore, X11s are thought to mediate a number of cellular functions through their association with various proteins [35]. This report showed that mutant mice lacking both X11 and X11L present with dysfunctional HCN1 channel activity and epileptic seizures. Notably, AD patients are also at increased risk of epileptic seizures. Furthermore, mutant mice lacking HCN1 demonstrated increased generation of Aβ, which is a causative factor for the development of AD.

The incidence of AD dramatically increases with age. We found that the amount of HCN1 decreased with both aging and in AD. The dysfunction of HCN1 that occurs over time may, thus, be a trigger for epileptic seizures and the pathogenic generation of Aβ in AD.

Several converging studies corroborate the premise that HCN channel activity is closely related to epileptogenesis [11]. For example, HCN1 expression is significantly reduced in the EC after temporal lobe epilepsy [36,37]. Furthermore, HCN1 channel plasticity in cortical neurons is similar in multiple epileptic animal models [38-43]. Moreover, kainic acid-induced seizure susceptibility is increased in HCN1^-/-^ mice [27], and HCN2-deficient mice exhibit spontaneous absence seizures [26].

HCN1^-/-^ mice show a significantly higher number of negative resting membrane potentials and a significantly higher input resistance measured from responses to either negative or positive current steps [28]. As such, seizure susceptibility is increased in HCN1^-/-^ mice [27], indicating that loss of the HCN1 subunit enhances neuronal excitability, which can increase Aβ generation [8-10]. These observations suggest that enhanced Aβ generation in HCN1^-/-^ mice results from neuronal hyper-excitability, which is in turn caused by ablation of the HCN1 gene.

On the other hand, the present study showed that HCN1 physically associated with APP through the extracellular domain of APP. Therefore, HCN1-mediated regulation of Aβ generation may depend on a molecular linkage between HCN1 and APP and not simply on alterations in neuronal excitability. However, the molecular mechanism by which HCN1 potentially links epileptic seizures to Aβ generation in AD remains to be elucidated.

Our results suggest that ablation of X11/X11L induces aberrant HCN1 distribution and function along with epilepsy. Although the molecular mechanism by which X11s regulates HCN channel activity also remains unclear, X11s are known to modulate intracellular trafficking of membrane proteins. For example, X11s interact with certain proteins implicated in traffic and transport, such as Arfs, Rab6, and KIF17 [29,44,45]. Furthermore, X11s bind to vesicular cargo proteins, such as APP and alcadein [16,46,47] and regulate the intracellular distribution of APP [21,30]. We hypothesize that X11 and X11L similarly influence the trafficking and/or intracellular localization of HCN1. We further hypothesize that the mislocalization of HCN1 observed in X11^-/-^/X11L^-/-^ mice (Additional file 1: Figure S4) may cause aberrant excitatory neuronal activities, resulting in epileptic seizures.

In conclusion, this study indicates that HCN1 may play an important role in the regulation of neuronal activity, along with Aβ generation in the hippocampal formation. However, we cannot rule out the possibility that additional ion channels (e.g., M-channels, Kir-channels, and sodium leak channels) also participate in the regulation of APP metabolism. Taken together, the current observations may provide new insights into the mechanisms underlying the linkage between epileptic seizures and Aβ generation in AD.

## Methods

### Animals and human non-AD and AD brain samples

All animal studies were conducted in compliance with the guidelines of the Animal Studies Committees of Hokkaido University (Sapporo, Japan), Shiga University (Shiga, Japan), and the National Institute of Biomedical Innovation (Osaka, Japan). Mice were maintained under a 12-h light/12-h dark cycle (lights on, 7:00 A.M.–7:00 P.M.), and provided with food and water *ad libitum*. X11^-/-^/X11L^+/+^, X11^+/+^/X11L^-/-^, and X11^-/-^/X11L^-/-^ mice have already been described [20,21]. HCN^-/-^ mice (stock number 005034) were purchased from The Jackson Laboratory (Bar Harbor, Maine). Male mice were used for all experiments.

Brain samples containing the superior temporal gyrus of cynomolgus monkeys (*Macaca fascicularis*) were obtained from Shiga University of Medical Science and the National Institute of Biomedical Innovation. The monkeys were housed in individual cages prior to the experiment and were maintained according to institutional guidelines for experimental animal welfare. Human brain samples containing the superior temporal gyrus (Broadmann area 22) were obtained from the Brain Bank for Aging Research, the Tokyo Metropolitan Institute of Gerontology (Itabashi, Tokyo, Japan). Human temporal cortical specimens for the quantification of proteins were obtained from brains that were removed, processed, and stored at −80°C within 16 h postmortem at the Brain Bank at Tokyo Metropolitan Institute of Gerontology. (Patients were placed in a cold (4°C) room within 2 h of death.) For all brains registered at the bank, written informed consent for their use for medical research was obtained from the patient prior to death or from the patient's family. Brain specimens were collected from Broadmann area 22 (superior temporal gyrus) for 12 AD patients (79.2±4.4 years of age) and 13 control patients (80.4±4.2 years of age) [48]. Detailed descriptions of all subjects, including the relative protein/tubulin ratio for each individual, are shown in Table 1.

### Antibodies

Polyclonal rabbit anti-HCN1 antibody [25] and polyclonal rabbit anti-X11 UT153 antibody [21] have already been described. Monoclonal mouse anti-tubulin DM1A antibody and polyclonal rabbit anti-c-Fos, rabbit anti-Egr-1, and goat anti-HCN1 antibodies (sc-19706) were purchased from Santa Cruz Biotechnology (Santa Cruz, CA, USA). Characterization and demonstration of the antigen-specificity of the goat anti-HCN1 antibody (sc-19706) is shown in Additional file 1: Figure S7. Monoclonal mouse anti-X11L/mint2 and anti-PSD95 antibodies were purchased from BD Transduction Laboratories (Lexington, KY, USA). Anti-actin antibody and the anti-HCN1 antibody, AB5884, were purchased from Millipore (Billerica, MA, USA). Anti-FLAG M2 and polyclonal rabbit anti-APP cytoplasmic domain (N-terminus) antibodies were purchased from Sigma-Aldrich (St. Louis, MO, USA), and the anti-human APP extracellular domain antibody (LN27) was purchased from Zymed (San Francisco, CA, USA). Anti-FLAG M2 affinity gel and FLAG peptide were purchased from Sigma-Aldrich.

### Plasmid construction

Human APP695 (hAPP695) and FLAG-APP695 cDNA were inserted into the pcDNA3 plasmid at the HindIII/XbaI restriction sites to produce pcDNA3-hAPP695 and pcDNA3-FLAG-hAPP695 [46]. The cDNA constructs pcDNA3-hAPP_Δcyt_ (in which amino acids 652–695 of hAPP695 are deleted) and pcDNA3.1-C99-FLAG (in which the signal sequence of hAPP is inserted into the 5’ region of C99) were generated by PCR using pcDNA3-hAPP695 as the template. The generated fragments were ligated into pcDNA3-hAPP695 and pcDNA3.1-FLAG at the BamHI/XbaI restriction site and the HindIII/XbaI restriction site, respectively. The pCI-*murine* HCN1 vector was a kind gift from Dr. Takahiro M. Ishii [49].

### Immunohistochemistry

Murine brain tissue sections were prepared and incubated with primary antibodies as described [21]. The sections were further incubated with goat anti-rabbit IgG antibodies conjugated to biotin (Vector Laboratories, Burlingame, CA, USA), followed by the ABC complex. Peroxidase activity was revealed using diaminobenzidine as the chromogen. Alternatively, sections were incubated with donkey anti-mouse IgG coupled with Alexa Fluor 488, donkey anti-rabbit IgG coupled with Cy3, or donkey anti-goat IgG coupled with Alexa Fluor 633 in phosphate buffered saline (PBS) containing 3% bovine serum albumin (BSA) for 2 h at room temperature. Sections were mounted onto slides with Shandon Immu-Mount (Thermo, Pittsburgh, PA, USA) and viewed under a BZ-9000 microscope (Keyence, Woodcliff Lake, NJ, USA).

### Immunoblotting and co-immunoprecipitation analysis

The cortices of wild type, X11^-/-^/X11L^+/+^, X11^+/+^/X11L^-/-^, X11^-/-^/X11L^-/-^, HCN1^+/-^, and HCN1^-/-^ mice, and cynomolgus monkey brains (superior temporal gyrus) and human post-mortem brains (superior temporal gyrus) were homogenized in eight volumes of radioimmune precipitation assay buffer containing 0.5% (w/v) sodium dodecyl sulfate (SDS) and a protease inhibitor mixture (5 μg/ml chymostatin, 5 μg/ml leupeptin, and 5 μg/ml pepstatin). The homogenates were lysed by sonication on ice and centrifuged at 20,000 × g for 10 min at 4°C. The resulting supernatants were used for immunoblot analysis. Proteins (10 μg per lysate) were separated via SDS (w/v) polyacrylamide gel electrophoresis (SDS-PAGE) on 7.5% (w/v) polyacrylamide gels.

The cortex (from one mouse) and EC-rich region (from five mice) from wild type and gene-null mice were homogenized in eight volumes of HBS-T lysis buffer (10 mM HEPES [pH 7.6] containing 150 mM NaCl, 5 mM EDTA, 0.5% [v/v] Triton X-100, 5 μg/ml chymostatin, 5 μg/ml leupeptin, and 5 μg/ml pepstatin A). Homogenates were then centrifuged at 20,000 × g for 10 min at 4°C. N2a cells (~1 × 10^6^) were transiently transfected with 0.8 μg pcDNA3-FLAG-hAPP695, pcDNA3-hAPP695, pcDNA3-hAPP_Δcyt_, or pcDNA3.1-C99-FLAG and 0.4 μg of pCI-*murine* HCN1 using Lipofectamine 2000 (Invitrogen) and cultured for 24 h in medium (DMEM) containing 10% (v/v) fetal bovine serum (FBS). Cells were harvested, lysed in lysis buffer (PBS containing 1.0% [v/v] Triton X-100, 5 μg/ml chymostatin, 5 μg/ml leupeptin, and 5 μg/ml pepstatin A), and centrifuged for 5 min at 4°C. The resulting supernatants were incubated with anti-FLAG M2, anti-hAPP extracellular domain (LN27), anti-HCN1, anti-X11, or anti-X11L/Mint2 antibody at 4°C for 2 h. Each immunocomplex was recovered with Dynabeads® Protein G (Invitrogen) and washed three times with lysis buffer. The proteins were separated on 7.5% (w/v) polyacrylamide gels, transferred onto nitrocellulose membranes, and analyzed by immunoblotting with the indicated antibodies. The immunoreactants were detected using the ECL plus™ detection system (GE Healthcare, Houston, TX, USA) and quantified using a Versa Doc model 3000 (Bio-Rad, Hercules, CA, USA).

### Affinity purification of FLAG-sAPP from N2a conditioned medium and immunoprecipitation of APP-HCN1 complex

N2a cells (~8.8 × 10^6^) were transiently transfected with 5 μg pcDNA3-FLAG-hAPP695 using Lipofectamine 2000 (Invitrogen) and cultured for 24 h in 8 mL of medium (DMEM) containing 10% (v/v) FBS. FLAG-sAPP was collected from the conditioned culture medium by using 50 μL of anti-FLAG M2 affinity gel. The collected FLAG-sAPP that was bound to the gel was washed twice with wash buffer I (20 mM Tris–HCl [pH 8.0], 1 M NaCl, and 0.1% Triton X-100) and twice with wash buffer II (50 mM Tris–HCl [pH8.0], 150 mM NaCl, 1% Triton X-100, 0.05% SDS, and 5 mM EDTA). Collected FLAG-sAPP was then eluted from the affinity gel with 20 μg FLAG-peptide and subjected to immunoblotting and Coomassie brilliant blue (CBB) staining to ascertain the degree of purification.

FLAG-sAPP or FLAG-peptide coupled to anti-FLAG M2 affinity beads were then incubated for 2 h at 4°C with HBS-T-soluble lysates derived from wild type N2a cells or N2a cells transiently overexpressing HCN1. The beads were washed three times with HBS-T lysis buffer. The proteins bound to the beads were separated on 7.5% (w/v) polyacrylamide gels, transferred onto membranes, and analyzed by immunoblotting with the indicated antibodies. The immunoreactants were detected using the ECL plus™ detection system (GE Healthcare) and quantified using a Versa Doc model 3000 (Bio-Rad).

### Quantification of Aβ40 and Aβ42

Endogenous murine Aβ was measured as described previously [21] using cortices dissected from 4-month-old mice. Murine Aβ40 and Aβ42 were measured using a sandwich ELISA (sELISA) system (mouse/rat Aβ40 and Aβ42 assay kit, Immuno-Biological Laboratories (IBL), Fujioka, Japan). N2a cells (~2 × 10^5^) were transiently transfected with 0.2 μg pcDNA3-FLAG-hAPP695 and 0.1 μg pCI-*murine* HCN1 using Lipofectamine 2000 (Invitrogen) and cultured in medium (DMEM) containing 10% (v/v) FBS. After 24 h, cells were incubated in fresh medium for an additional 4 h with or without 10 μM ZD7288 (Tocris Bioscience, Bristol, UK). Human Aβ40 (hAβ40) and hAβ42 secreted into the culture medium during the 4-h incubation were quantified using the sELISA system.

### Electroencephalogram recording

To obtain free-moving cortical electrocorticogram recordings, recording and reference electrodes were screwed onto the skull over the temporal (anterior = −3.1 mm, lateral = 2.5 mm, relative to bregma) and occipital regions of the murine brain. Recordings were continuously made using a cortical electroencephalogram linked to a telemetry system (Unimec, Usmate Velate, Italy) throughout the experiment [50].

### Ih current recording

All experiments were performed in a blinded manner. Mice (12–14 weeks old) were anesthetized with halothane (Takeda Chemical Industries) and then sacrificed by decapitation. The brain was rapidly removed and immediately placed in a cold (4°C) cutting solution, which contained 234 mM sucrose, 2.5 mM KCl, 1.1 mM NaH_2_PO_4_, 10 mM MgSO_4_, 26 mM NaHCO_3_, 12 mM glucose, and 0.5 mM CaCl_2_. Horizontal slices (300 μm thick), which included the EC and the hippocampus, were prepared using a vibratome (VT1000S, Leica, Nussloch, Germany). During recording, individual slices were transferred to a submerged recording chamber and continuously perfused with artificial cerebrospinal fluid (ACSF) maintained at 30–32°C. The ACSF contained 125 mM NaCl, 2.5 mM KCl, 1.1 mM NaH_2_PO_4_, 1.0 mM MgSO_4_, 26 mM NaHCO_3_, 12 mM glucose, and 2.0 mM CaCl_2_ and was saturated with 95% O_2_ and 5% CO_2_. Whole-cell patch-clamp recordings were obtained from principal excitatory cells in layer II of the EC. The patch pipettes were filled with an intracellular solution containing 30 mM K-methanesulfonate, 6 mM NaCl, 0.2 mM EGTA, 10 mM HEPES, 4 mM Mg-ATP, 0.3 mM Na_3_-GTP, and 10 mM phosphocreatine-Tris (pH 7.3). In layer II cells of the EC, the hyperpolarization-induced and “slowly-activating” inward currents in the voltage-clamp mode mainly consisted of Ih currents [51]. When Ih currents were studied in the voltage-clamp mode, membrane potentials were first held at −65 mV, and then voltage steps with a duration of 7 s were applied from −55 mV to −125 mV (10 mV increments), after which the holding potentials were allowed to return to −65 mV to obtain the tail currents. The amplitudes of the tail currents at 50 ms after the end of the final voltage step were analyzed to obtain the Ih currents. In all electrophysiological analyses, pooled data were represented as the mean ± SEM.

### Statistical analysis

Statistical analyses were performed using a two-tailed Mann–Whitney *U*-test, a one-way analysis of variance followed by Tukey’s multiple comparison test, or the two-tailed Pearson's correlation coefficient. All analyses were conducted with GraphPad Prism 5 software.

## Abbreviations

ACSF: Artificial cerebrospinal fluid; AD: Alzheimer’s disease; APP: Amyloid precursor protein; Aβ: Amyloid β peptide; DG: Dentate gyrus; EC: Entorhinal cortex; FBS: Fetal bovine serum; HCN channel: Hyperpolarization-activated cyclic nucleotide gated channel; Ih current: Hyperpolarization-activated current; PBS: Phosphate buffered saline; sAPP: Soluble APP; SDS: Sodium dodecyl sulfate; sELISA: Sandwich ELISA; SDS-PAGE: SDS polyacrylamide gel electrophoresis; TBS: Tris buffere saline; X11L: X11-like; X11L2: X11-like2; X11s: X11 proteins.

## Competing interests

The authors declare no competing interests.

## Authors' contributions

YS, TI, GZ, MO, KI, SK and TS generated the hypotheses for the mouse, monkey and human projects. YS and TS drafted the manuscript. YS, TI, GZ, MO, MN, SK, RS, KI, and TS edited the manuscript and contributed to discussion. YS and NK performed the biochemical and histochemical analyses for the mouse and monkey studies. YS, MN and SM performed biochemical analyses for the human study. TI and KI conducted electrophysiological analyses for the mouse study. YS, GZ, MO and SK performed electroencephalogram recordings. MN and NK provided monkey tissues, and SM provided human tissues. All authors read and approved the final manuscript.

## Supplementary Material

Additional file 1 **Figure S1.** Simultaneous recording of electrocorticogram in epilepsy model mice and corresponding movie. A representative electrocorticogram recorded during the interictal period in 13-week-old X11^-/-^/ X11L^-/-^ mice (n = 4) is shown. The underlined region indicates the time frame of the corresponding movie (Movie S3). **Figure S2**. Individual data of Ih currents density in entorhinal cortex layer II neurons of wild-type and X11s-null mice. (***A***) Indicidual data of Ih current density. Blue indicate the data of mouse #1 and red indicate mouse #2. (***B***) Mean, SD, SEM, and count number of *A.* P Value of Student’s t-test (#1 vs #2) shown in bottom line. (***C***) Distribution and average of current density of *A*. Closed symbols indicate the data of mouse #1 and opened symbols indicate mouse #2 (mean ± SEM). **Figure S3**. HCN1 levels in the EC-rich region of the brains of X11^+/+^/X11L^+/+^ and X11s mutant mice. (***A***) Isolation of the EC-rich region from a horizontal slice (300 μm thick) of murine brain. Brain slices from 13-week-old X11^+/+^/X11L^+/+^, X11^+/+^/X11L^-/-^, X11^-/-^/X11L^+/+^, and X11^-/-^/X11L^-/-^ mice were prepared in ice-cold PBS using a vibratome (VT1200S; Leica) (left panel). The EC-rich region (EC) was separated from each slice as indicated (right panel). (***B, C***) Quantification of HCN1 in the EC-rich region. Horizontal slices were homogenized in eight volumes of radioimmune precipitation assay buffer containing 0.5% (w/v) SDS and a protease inhibitor mixture (5 μg/ml chymostatin, 5 μg/ml leupeptin, and 5 μg/ml pepstatin), subjected to sonication on ice, and centrifuged at 20,000 × g for 10 min at 4°C. (*B*) The resulting supernatants (each containing 10 μg protein) were analyzed by SDS-PAGE on 7.5% (w/v) polyacrylamide gels, followed by immunoblotting with anti-HCN1, anti-X11, anti-X11L, and anti-tubulin antibodies (n = 4). (*C*) The HCN1 level was normalized to the tubulin level to give the relative HCN1/tubulin ratio for each genotype (mean ± SEM, n = 4). **Figure S4**. Altered distribution of the HCN channel in X11^-/-^/X11L^-/-^ mice. (***A***) Low-power images of horizontal brain sections from 13-week-old wild type (X11^+/+^/X11L^+/+^: *upper panels*) and X11^-/-^/X11L^-/-^ (*Lower panels*) mutant mice were immunostained with an anti-HCN1 antibody (n=3). Scale bar, 300 μm. (***B***) Representative high-resolution images of horizontal brain sections from 13-week-old X11^+/+^/X11L^+/+^ (*a, c*) and X11^-/-^/X11L^-/-^ (*b, d*) mice were subjected to immunostaining with an anti-HCN1 antibody (*a, b*) and Nissl stain (*c, d*). (***C***) Quantitative analysis of HCN1 immunoreactivity in the EC of 13-week-old wild type (X11^+/+^/X11L^+/+^) and X11^-/-^/X11L^-/-^ mutant mice. The intensity of the HCN1 immunoreactivity in the areas enclosed by the open boxes in *A* was measured using NIH Image J software. Scale bar, 50 μm. **Figure S5**. Complex formation of HCN1 with APP in N2a cells treated with ZD7288. FLAG-APP and HCN1 were transiently overexpressed in N2a cells (~1 × 10^6^) with (+) or without (−) 10 μM ZD7288. To standardize the amount of plasmid transfected into the cells, an empty vector (−) was added to yield 1.2 μg of plasmid in total. The cell lysates were subjected to immunoprecipitation with anti-FLAG M2 antibody. Immunocomplexes were detected by immunoblotting with anti-HCN1 and anti-FLAG antibodies. **Figure S6**. Covariance analysis of various protein levels in the brain of cynomolgus monkeys. (*A–H*) Levels of HCN1, APP, Aβ40, and Aβ42 in the brain (superior temporal gyrus) of 4–37-year-old cynomolgus monkeys were quantified by immunoblotting and sELISA assay. Protein levels were normalized to tubulin levels or to tissue weight to give the relative protein/tubulin ratio for immunoblotting and the relative protein/tissue weight ratio for sELISA. (***A***) Correlation between age and APP level (n = 39, r = 0.8156, ****p < 0.0001). (***B***) Correlation between HCN1 and APP levels (n = 39, r = −0.3796, **p = 0.0086). (***C, D***) Correlation between HCN1 and Aβ40 levels (n = 39, r = −0.2878, *p = 0.0421). An enlarged view of (*C*) in the 0 to 1,000 *f* mol/mg tissue range is shown in (*D*) (***E, F***) Correlation between HCN1 and Aβ42 levels (n = 39, r = −0.2913, *p = 0.0401). An enlarged view of (*E*) in the 0 to 400 *f* mol/mg tissue range is shown in (*F*). (***G***) Correlation between APP and Aβ40 (n = 39, r = 0.2993, *p* = 0.072). (***H***) Correlation between APP and Aβ42 levels (n = 39, r = 0.3714, *p = 0.0236). Statistical analysis was performed using the two-tailed Pearson's correlation coefficient. **Figure S7**. Specificity of the polyclonal goat anti-HCN1 antibody. (***A***) Competition analysis using glutathione-S-transferase (GST) fused to the 60-amino acid carboxyl terminal region of murine HCN1 (mHCN1 C60). This region of the protein contains the epitope for the goat anti-HCN1 antibody used in this study (sc-19706: Santa Cruz Biotechnology). Brain lysates (10 μg protein) derived from HCN1^+/+^ and HCN1^-/-^ mice and cynomolgus monkeys were subjected to immunoblot analysis. The anti-HCN1 antibody was pre-incubated with 20 μg GST alone or GST-mHCN1 C60 recombinant protein at 4^o^C for 2 h. The pre-incubated antibody was then reacted with the immunoblots. HCN1 was detected in HCN1^+/+^ mouse and monkey brains when the antibody was pre-incubated with GST alone, but not when the antibody was pre-incubated with GST-mHCN1 C60. (***B***) Titer comparison between anti-HCN1 antibodies. Brain lysates (10 μg protein) were subjected to immunoblot analysis with two commercial anti-HCN1 antibodies (sc-19706, Santa Cruz Biotechnology; and AB5884, Millipore). (***C***) Specificity of goat anti-HCN1 antibody (sc-19706) for immunohistochemical analysis. (***a, b***) Representative images of horizontal brain sections showing the hippocampal formation in 13-week-old HCN1^+/+^ (*a*) and HCN1^-/-^ (*b*) mice stained with goat anti-HCN1 antibody, followed by donkey anti-goat IgG coupled with FITC. (***c, d***) Magnified view of the squares in (***a***) and (***b***). HCN1 signals (green) observed in HCN1^+/+^ mice were absent in HCN1^-/-^ mice. Nuclei counter-stained with DAPI are shown in blue. Scale bars, 300 μm (*a, b*), 50 μm (*c, d*). Click here for file

Additional file 2 **Movie S1.** Spontaneous epileptic seizures in X11^-/-^/X11L^-/-^ mice. The electrocorticogram of Supplementary Figure S1 and Movie S3 were simultaneously recorded. Click here for file

Additional file 3 **Movie S2.** Spontaneous epileptic seizures in X11^-/-^/X11L^-/-^ mice. The electrocorticogram of Supplementary Figure S1 and Movie S3 were simultaneously recorded. Click here for file

Additional file 4 **Movie S3.** Spontaneous epileptic seizures in X11^-/-^/X11L^-/-^ mice. The electrocorticogram of Supplementary Figure S1 and Movie S3 were simultaneously recorded. Click here for file
