# Supplementary material for: Hyperpolarization-activated cyclic nucleotide gated channels: a potential molecular link between epileptic seizures and Aβ generation in Alzheimer’s disease
Source: Mol Neurodegener. 2012 Oct 3;7:50. doi: 10.1186/1750-1326-7-50 (PMC3524764; doi:10.1186/1750-1326-7-50)

Supplementary Fig. S1

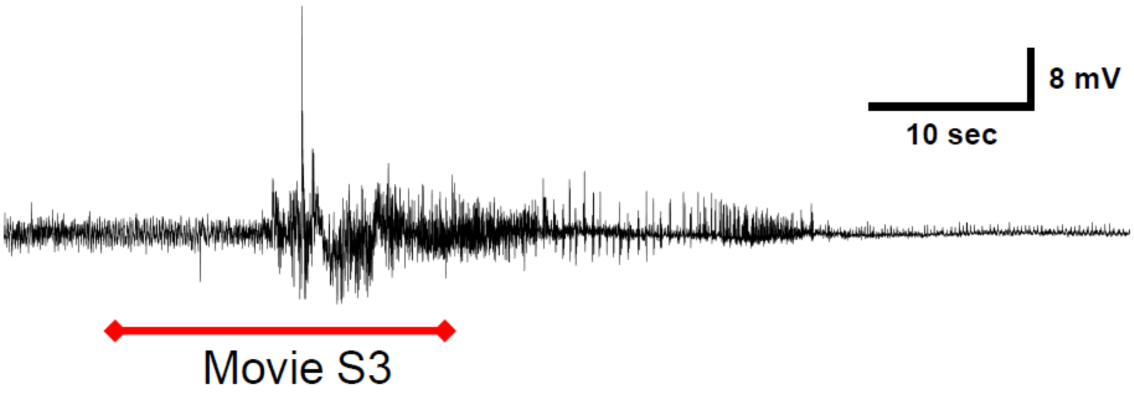

Supplementary Fig. S2

A

	current density (pA/pF)				
	Cell #	X11+/+ X11L+/+	X11−/− X11L+/+	X11+/+ X11L−/−	X11−/− X11L−/−
Mouse #1	1	2.75	2.56	3.36	1.17
	2	2.28	1.27	1.86	0.93
	3	1.11	3.60	2.52	0.79
	4	1.09	2.38	1.76	1.26
	5	2.10	2.96	2.72	1.19
Mouse #2	6	1.17	2.64	2.91	0.80
	7	2.92	1.26	1.01	0.46
	8	1.88	2.55	0.80	1.92
	9	3.12	2.51	1.55	1.55
	10	3.38			

B

#1 plus #2				
	X11+/+ X11L+/+	X11−/− X11L+/+	X11+/+ X11L−/−	X11−/− X11L−/−
Mean	2.18	2.41	2.05	1.12
SD	0.86	0.75	0.88	0.44
SEM	0.27	0.25	0.29	0.15
Count	10	9	9	9
**#1**				
Mean	1.87	2.55	2.45	1.07
SD	0.74	0.86	0.66	0.20
SEM	0.33	0.38	0.29	0.09
Count	5	5	5	5
**#2**				
Mean	2.50	2.24	1.57	1.18
SD	0.94	0.65	0.95	0.67
SEM	0.42	0.33	0.47	0.33
Count	5	4	4	4
**t−test (#1 vs #2)**				
P Value	0.27	0.57	0.14	0.72

C

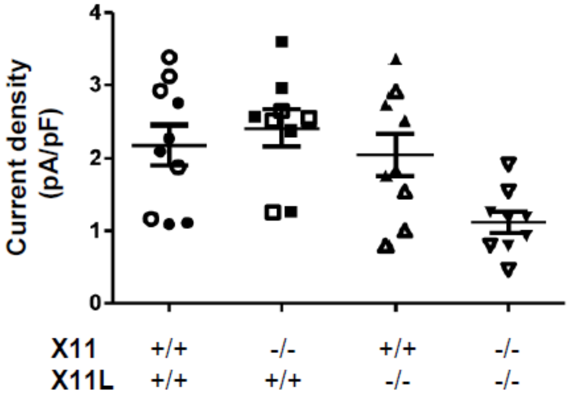

Supplementary Fig. S3

A

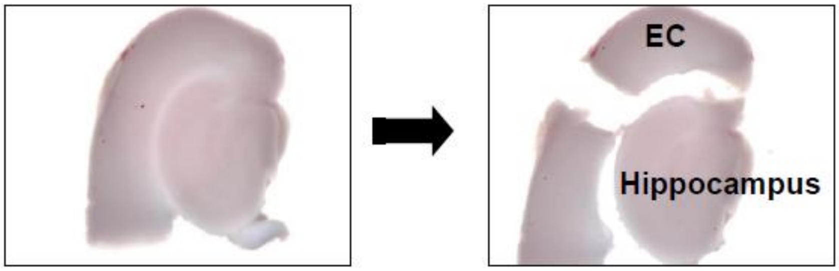

B

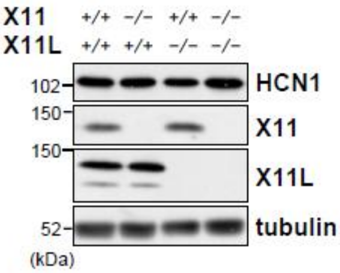

C

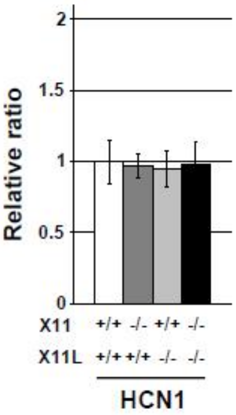

Supplementary Fig. S4

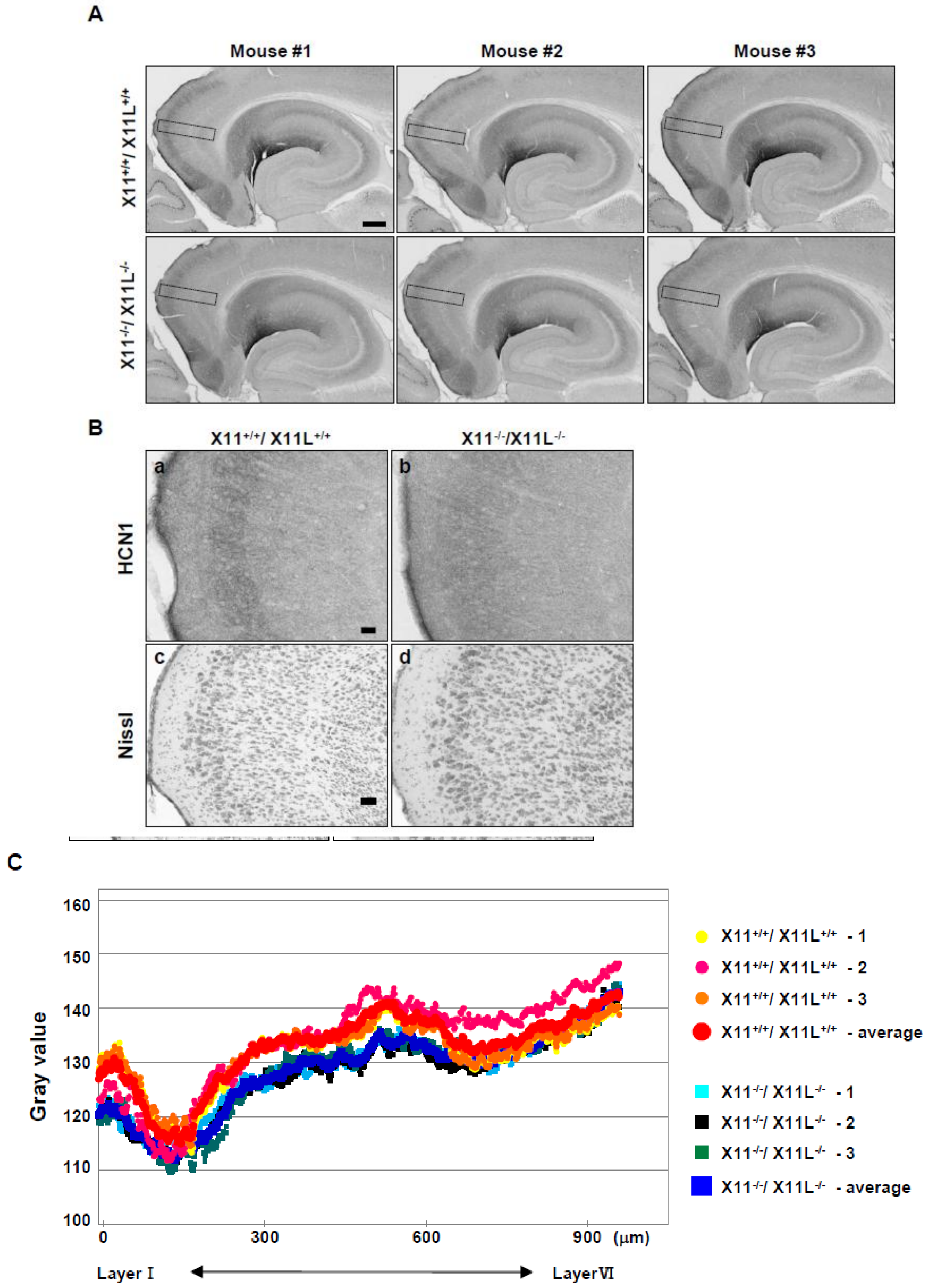

Supplementary Fig. S5

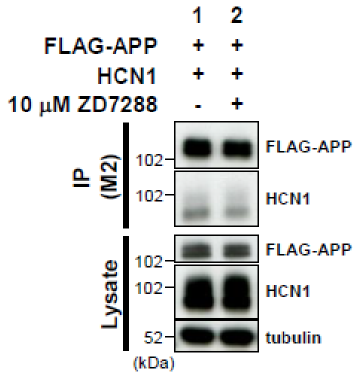

Supplementary Fig. S6

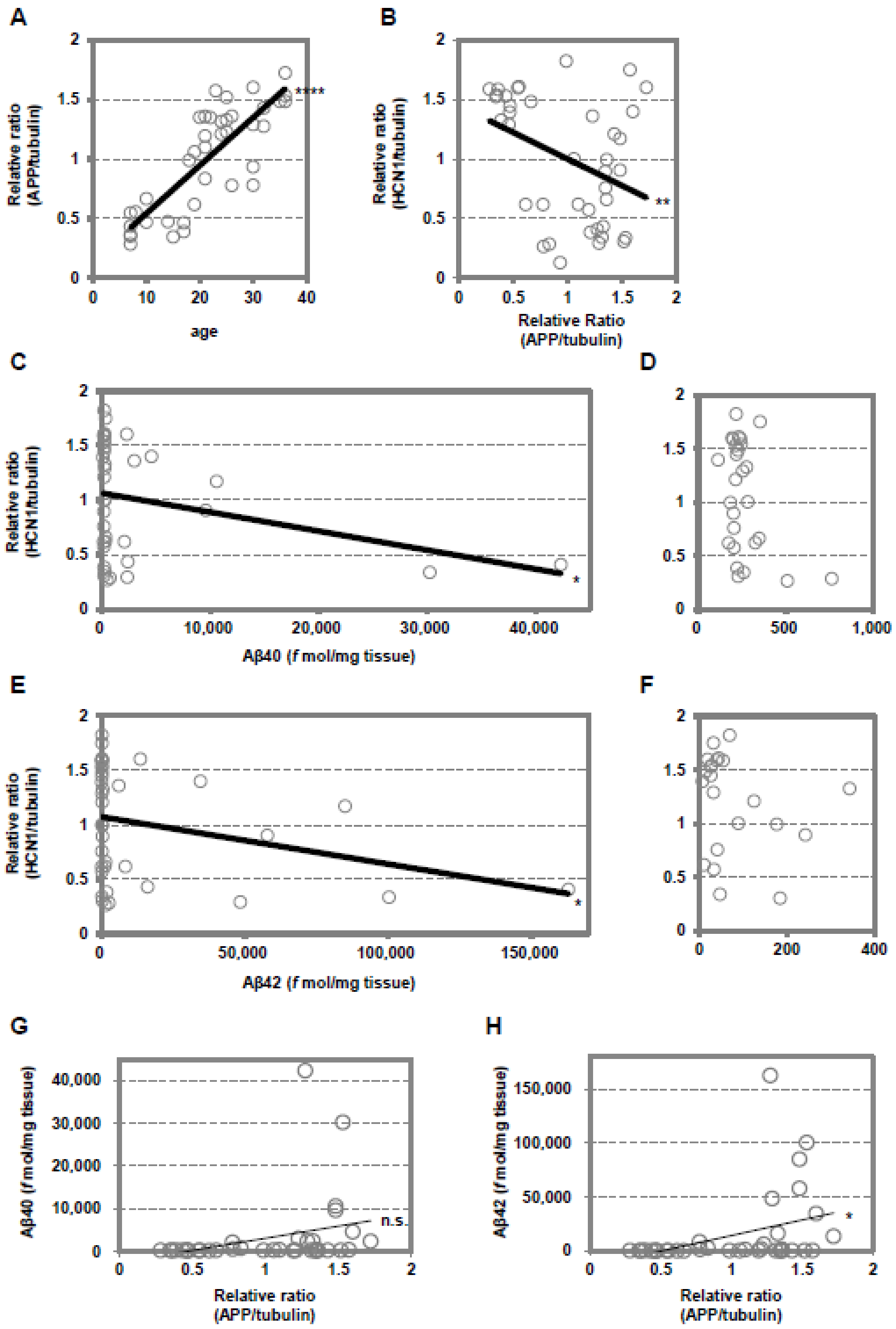

Supplementary Fig. S7

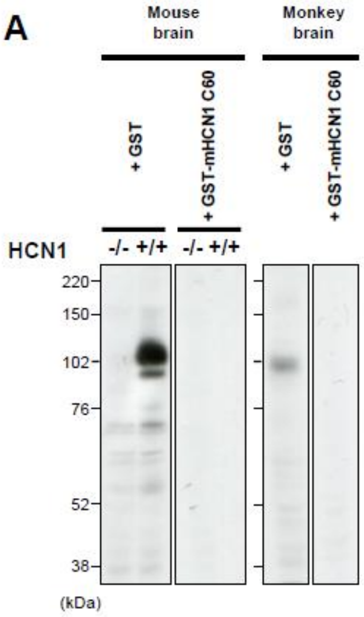

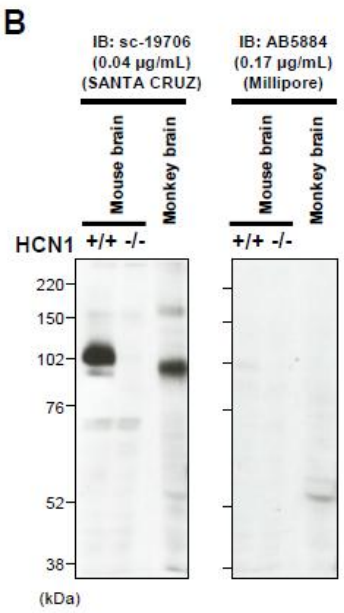

C

HCN1 +/+

HCN1 -/-

C

HCN1 +/+

HCN1 -/-

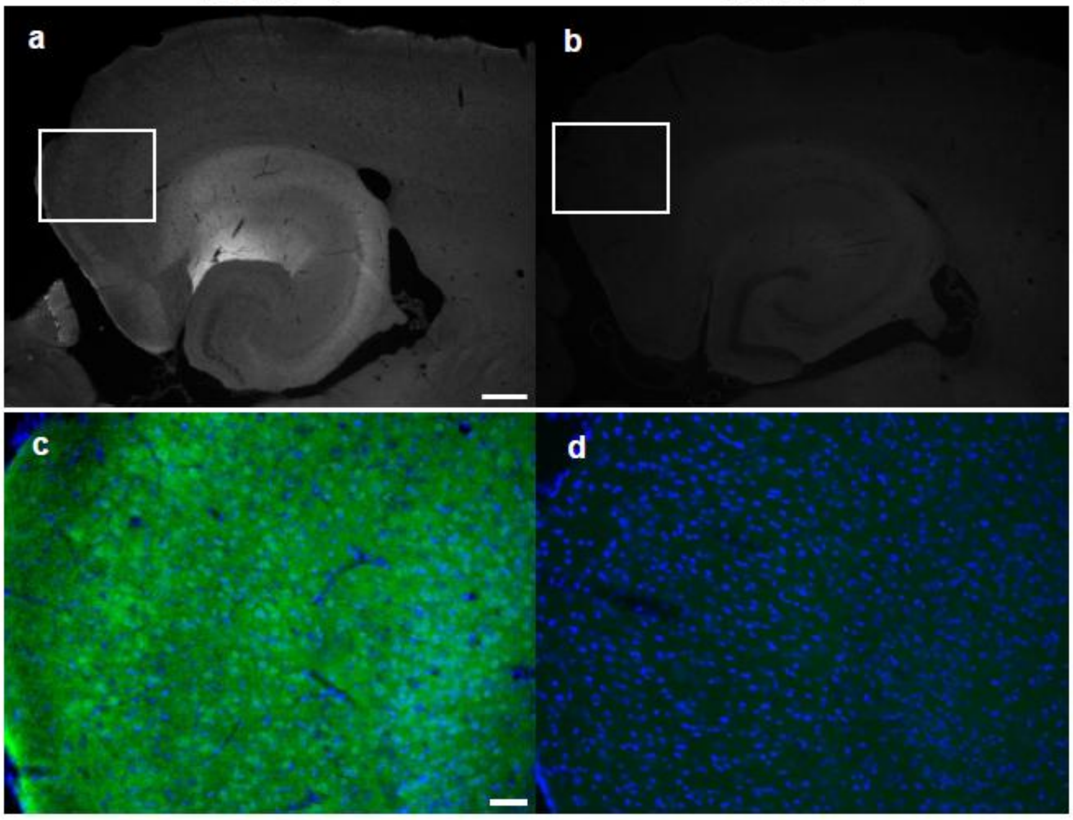

Supplement: Additional file 1 — Figure S1. Simultaneous recording of electrocorticogram in epilepsy model mice and corresponding movie. A representative electrocorticogram recorded during the interictal period in 13-week-old X11-/-/ X11L-/- mice (n = 4) is shown. The underlined region indicates the time frame of the corresponding movie (Movie S3). Figure S2. Individual data of Ih currents density in entorhinal cortex layer II neurons of wild-type and X11s-null mice. (A) Indicidual data of Ih current density. Blue indicate the data of mouse #1 and red indicate mouse #2. (B) Mean, SD, SEM, and count number of A. P Value of Student’s t-test (#1 vs #2) shown in bottom line. (C) Distribution and average of current density of A. Closed symbols indicate the data of mouse #1 and opened symbols indicate mouse #2 (mean ± SEM). Figure S3. HCN1 levels in the EC-rich region of the brains of X11+/+/X11L+/+ and X11s mutant mice. (A) Isolation of the EC-rich region from a horizontal slice (300 μm thick) of murine brain. Brain slices from 13-week-old X11+/+/X11L+/+, X11+/+/X11L-/-, X11-/-/X11L+/+, and X11-/-/X11L-/- mice were prepared in ice-cold PBS using a vibratome (VT1200S; Leica) (left panel). The EC-rich region (EC) was separated from each slice as indicated (right panel). (B, C) Quantification of HCN1 in the EC-rich region. Horizontal slices were homogenized in eight volumes of radioimmune precipitation assay buffer containing 0.5% (w/v) SDS and a protease inhibitor mixture (5 μg/ml chymostatin, 5 μg/ml leupeptin, and 5 μg/ml pepstatin), subjected to sonication on ice, and centrifuged at 20,000 × g for 10 min at 4°C. (B) The resulting supernatants (each containing 10 μg protein) were analyzed by SDS-PAGE on 7.5% (w/v) polyacrylamide gels, followed by immunoblotting with anti-HCN1, anti-X11, anti-X11L, and anti-tubulin antibodies (n = 4). (C) The HCN1 level was normalized to the tubulin level to give the relative HCN1/tubulin ratio for each genotype (mean ± SEM, n = 4). Figure S4. Altered distribution of the [file 1750-1326-7-50-S1.pdf]
